# Spectrum of Endocrine Dysfunction and Association With Disease Severity in Patients With COVID-19: Insights From a Cross-Sectional, Observational Study

**DOI:** 10.3389/fendo.2021.645787

**Published:** 2021-07-02

**Authors:** Liza Das, Pinaki Dutta, Rama Walia, Soham Mukherjee, Vikas Suri, Goverdhan Dutt Puri, Varun Mahajan, Pankaj Malhotra, Shakun Chaudhary, Rahul Gupta, Satyam Singh Jayant, Kanhaiya Agrawal, Vijay Kumar, Naresh Sachdeva, Ashu Rastogi, Sanjay Kumar Bhadada, Sant Ram, Anil Bhansali

**Affiliations:** ^1^ Department of Endocrinology, Post Graduate Institute of Medical Education and Research (PGIMER), Chandigarh, India; ^2^ Department of Internal Medicine, PGIMER, Chandigarh, India; ^3^ Department of Anaesthesia and Intensive Care, PGIMER, Chandigarh, India; ^4^ Department of Haematology, PGIMER, Chandigarh, India; ^5^ Department of Biochemistry, PGIMER, Chandigarh, India

**Keywords:** COVID-19, endocrinology, hormones, central hypoadrenalism, mixed thyroid dysfunction, hypogonadism

## Abstract

**Introduction:**

Evidence on new-onset endocrine dysfunction and identifying whether the degree of this dysfunction is associated with the severity of disease in patients with COVID-19 is scarce.

**Patients and Methods:**

Consecutive patients enrolled at PGIMER Chandigarh were stratified on the basis of disease severity as group I (moderate-to-severe disease including oxygen saturation <94% on room air or those with comorbidities) (n= 35) and group II (mild disease, with oxygen saturation >94% and without comorbidities) (n=49). Hypothalamo-pituitary-adrenal, thyroid, gonadal axes, and lactotroph function were evaluated. Inflammatory and cell-injury markers were also analysed.

**Results:**

Patients in group I had higher prevalence of hypocortisolism (38.5 *vs* 6.8%, p=0.012), lower ACTH (16.3 *vs* 32.1pg/ml, p=0.234) and DHEAS (86.29 *vs* 117.8µg/dl, p= 0.086) as compared to group II. Low T3 syndrome was a universal finding, irrespective of disease severity. Sick euthyroid syndrome (apart from low T3 syndrome) (80.9 *vs* 73.1%, p= 0.046) and atypical thyroiditis (low T3, high T4, low or normal TSH) (14.3 *vs* 2.4%, p= 0.046) were more frequent in group I than group II. Male hypogonadism was also more prevalent in group I (75.6% *vs* 20.6%, p=0.006) than group II, with higher prevalence of both secondary (56.8 *vs* 15.3%, p=0.006) and primary (18.8 *vs* 5.3%, p=0.006) hypogonadism. Hyperprolactinemia was observed in 42.4% of patients without significant difference between both groups.

**Conclusion:**

COVID-19 can involve multiple endocrine organs and axes, with a greater prevalence and degree of endocrine dysfunction in those with more severe disease.

## Introduction

The pandemic of COVID-19, caused by SARS-CoV-2, has established itself as one of the greatest challenges to healthcare globally. Despite the lower case fatality rate as compared to other coronaviruses, COVID-19 has underscored the vulnerability of certain subsets of populations to emerging diseases, particularly with respect to gender, age, and co-morbidities (especially diabetes, hypertension, and obesity) (1–3). The hormonal basis of most of these underlying risk factors is undeniable, highlighting a crucial link between pre-existing metabolic conditions and predisposition to adverse outcomes with COVID-19 (4–7). On the other hand, the fact that SARS-CoV-2 can cause new-onset endocrine dysfunction is being increasingly recognized, with connotations extending to mortality (association with high cortisol) and possible therapeutic interventions (dexamethasone use in the RECOVERY trial) (8, 9).

The basis of endocrine dysfunction in COVID-19 can be attributable to organ-specific ACE2 mediated viral entry and damage, direct viral toxicity, and a ‘cytokine storm’ mediated by various cytokines (IL-6, IL-10, IL-1β, IL-17, and TNF-α) (10). ACE2, the entry receptor for SARS-CoV-2, has been identified in several endocrine organs like the thyroid, testes, pancreatic islets, and adipose tissue (11). Interestingly, the thyroid, testes, and adipose tissue have higher demonstrable levels of ACE2 than in the lungs. Others, like the hypothalamus, pituitary, and adrenals, have been found to harbor low expression of this receptor. Further, the transmembrane serine protease TMPRSS2, which acts as a co-receptor in viral entry by priming the spike protein of SARS-CoV-2, is also present in various endocrine organs, having the highest levels in the thyroid followed by the pancreas, pituitary, and testes (11).

However, overall clinical evidence of endocrine involvement in COVID-19 patients is limited (8, 12–20), mostly confined to case reports and small patient series (15–18, 20). Moreover, some of the patients in these reports had other disorders that could explain their presentation (including adrenal hemorrhage in a COVID-19 patient with APLA syndrome, death in a COVID-19 patient with myxoedema coma) (18, 20). The other available body of evidence is extrapolated from studies involving SARS patients, their autopsy data, and retrospective studies in SARS survivors (21–23). Though there is a genomic concordance between SARS-CoV and SARS-CoV-2 of almost 80% and ACE2 is the common host viral entry receptor for both, there is an inherent difference between infectivity and binding affinity to ACE2 between both viruses. In this context, caution needs to be exercised in the generalisability of clinical parameters and outcomes, and evidence pertaining to COVID-19 needs to be explored so as to improve patient management. Further, whether there is an association between disease severity and endocrine function in COVID-19 needs investigation.

This was a single-center, cross-sectional study to evaluate endocrine function in patients with COVID-19 and to investigate whether the hormonal function is similar or different between patients with mild (symptomatic or asymptomatic) and moderate-to-severe disease.

## Material and Methods

This was an observational study of consecutive patients with COVID-19 admitted at a single dedicated COVID care center at the Post Graduate Institute of Medical Education and Research (PGIMER), Chandigarh, India (n=74). Patients were diagnosed on the basis of RT-PCR positivity for SARS-CoV-2 in nasopharyngeal and/or throat swab samples. Their clinical, biochemical, and hormonal parameters were recorded. Clinical parameters included symptomatology type and duration and prior co-morbidities. Biochemical profiles included complete hemogram, renal and liver function tests, and inflammatory markers (CRP, procalcitonin, D-dimer, LDH, and ferritin). The neutrophil-lymphocyte ratio (NLR) and platelet-lymphocyte ratios (PLR) were calculated from the differential cell count. Patients were divided into two groups depending on disease severity and comorbidities: Group I [consisting of patients with moderate or severe disease defined as hypoxia with an inability to maintain oxygen saturation to at least 94% on room air (requiring high flow or conventional oxygen therapy or mechanical ventilation) or with chronic comorbidities, including diabetes, hypertension, malignancy, chronic liver or kidney disease, and chronic pulmonary disease] (n=35) and Group II (consisting of mild disease defined as the ability to maintain oxygen saturation to 94% or above at room air and without any chronic disease conditions) (n=49). Patients whose clinical data were not available were excluded from the study. The severity of disease for each patient was calculated as per CSS (scoring system of COVID-19), developed and validated previously for admitted patients with COVID-19 (24). This score has been found to be useful for predicting in-hospital complications, with good discriminatory ability (AUC 0.919), which is comparable to the APACHE-II and higher than the SOFA and CURB-65 scores in patients with COVID-19.

Hormonal parameters were assessed within 24 to 48 hours of admission in a sample taken at 0800 to 0900h and sent under appropriate cold-chain conditions. Serum cortisol, ACTH, DHEAS, free T3, free T4, TSH, Prolactin, LH, FSH, testosterone, and estradiol were estimated. For assessment of the hypothalamo-pituitary-adrenal (HPA) axis, patients who had been initiated on systemic glucocorticoid therapy for COVID-19 or on chronic glucocorticoid therapy for autoimmune or other conditions were excluded. Further, those with serum albumin<3g/dl were also excluded in order to eliminate falsely low cortisol concentrations since 10% of circulating cortisol is bound to albumin. For assessment of the hypothalamo-pituitary-thyroid (HPT) axis, patients already on thyroid hormone replacement or anti-thyroid drugs were excluded. There was no instance of exposure to iodinated contrast for CT scan and exposure to heparin was nil/minimal as samples were withdrawn within 24 hours (maximum 48 hours of admission). Hemogram, electrolytes, and liver and renal function tests were estimated by Beckman Coulter. All hormones and procalcitonin were estimated by electro-chemiluminescence assay (ECLIA, COBAS 8000, Roche Diagnostics, Germany) with a co-efficient of variation (CV) of 6% to 8%. HbA1c was measured by HPLC (Biorad variant II. CV 0.5 to 1%). CRP was measured by immunoturbidimetry, LDH by spectrophotometry, and ferritin by ECLIA.

The HPA axis status was defined based on the disease severity categorization. Cortisol was estimated in all patients by ECLIA, with the normal range of the assay being 170 to 550nmol/L (6 to 20µg/dl). Patients in Group I (with moderate-to-severe disease) were considered to be hypocortisolic if they had serum cortisol below 15µg/dl (414nmol/L) and eucortisolic with a level exceeding 15µg/dl (414nmol/L) (25). Patients in Group II (with mild disease or even asymptomatic) were considered hypocortisolic if they had serum cortisol levels below 6µg/dl (170nmol/L, lower limit of normal for the assay) and eucortisolic if their levels exceeded 6µg/dl (170nmol/L). Hypercortisolism was defined as cortisol levels exceeding 30µg/dl (900nmol/l) for group I and 20µg/dl (550nmol/l, upper limit of normal [ULN] of assay) for group II. Further subclassification was done depending on low/inappropriately normal ACTH (<65pg/ml, ULN) or high ACTH (>65pg/ml). DHEAS levels were classified on the basis of age- and gender-matched range. Regarding thyroid function tests, low T3 syndrome was defined as free T3 less than 2pg/ml (lower limit of normal [LLN] of the assay) (2pg/ml), and low T4 syndrome was defined as free T4 less than 0.9ng/dl (LLN), both irrespective of TSH. Sick euthyroid syndrome (SES) was defined either in the presence of low T3 or low T4 syndromes with suppressed or normal TSH as described above, or normal free T4 with low TSH. Thyrotoxicosis associated with typical thyroiditis was defined as elevated free T4 (>1.71ng/dl ULN), elevated free T3 (>4.4pg/ml, ULN), with a suppressed TSH (<0.4mIU/L). Hypothyroidism was defined as subclinical in the presence of normal free T4 with a concurrently raised TSH (4.2 and above) and overt in the presence of low free T4 with TSH exceeding 10mIU/L. Secondary hypogonadism was defined as testosterone (T) below the lower limit of normal (9nmol/l) with low or inappropriately normal gonadotropins (LH, FSH). Primary hypogonadism was defined as low testosterone with elevated gonadotropins. Gonadal status was evaluated and classified using standard criteria, as mentioned above, in male patients. In females, menopausal status was determined in those with levels exceeding the standard cutoff of 40mIU/ml. Hyperprolactinemia was defined as prolactin levels exceeding 20ng/ml in males and 25ng/ml in females, and hypoprolactinemia was defined as serum prolactin levels below 5ng/ml.

All study protocols were carried out in accordance with relevant guidelines and recommendations. The study was approved by the Institutional Ethics Committee, PGIMER (IEC/NK/6515/study/854).

### Statistical Analysis

Statistical analysis was performed using the Statistical Package for Social Sciences (SPSS) 22.0 software program (IBM Statistics 22.0). Qualitative variables were compared between the groups using the Pearson χ2 test or Fisher’s exact test. Quantitative variables were checked for normality using the Kolmogorov-Smirnov test and classified as parametric and non-parametric. The Student T-test was used to compare the means of two groups for parametric data and the Mann-Whitney U test for non-parametric data. Spearman correlation was used to determine the association between hormonal and biochemical parameters, with 0.3 to 0.5 being defined as moderate and >0.5 defined as strong correlation. A p-value <0.05 was considered significant.

## Results

There were a total of 84 patients enrolled in the study, of which 35 (41.7%) had moderate-to-severe disease (Group I) and 49 (58.3%) had mild disease (Group II). In Group I, 34.2% had hypoxia (defined as the inability to maintain O2 saturation of 94% at FiO2 of 0.2) and 77.1% had chronic comorbidities. Of these, hypertension was the most common (45.7%) followed by diabetes mellitus (33.3%), chronic airway disease (18.5%, including COPD, interstitial lung disease, chronic pulmonary aspergillosis, and cryptococcosis), chronic liver disease (18.5%), and CAD (11.1%). A single comorbidity was present in 51.8% of patients, and the rest had two or more chronic disease conditions; both hypertension and diabetes were present in 22.8% of patients. Diabetes mellitus was present in 33.3% of patients and the details of the medication of these patients were present in all 10 subjects. Only two patients were on insulin, and the rest were on oral antidiabetic agents, the most common being metformin followed by DPP-IV inhibitors (teneligliptin and linagliptin) and glimepiride, at the time of admission. CSS>2 was present in a significantly greater proportion of patients in group I. Baseline clinical and biochemical parameters were analyzed for all patients. Among hormonal axes, the HPA axis was analyzed in all except those who were initiated on systemic glucocorticoid therapy (n=7) and those with albumin below 3g/dl (n=11). The HPT axis was analyzed in all except four patients, who were already on thyroid hormone replacement for hypothyroidism. All four patients were on levothyroxine replacement (in doses varying from 50 to 100µg). Further, there were two patients with non-alcoholic fatty liver disease, one of whom presented with hematemesis and decompensated liver disease.

Baseline clinical parameters are summarised in **Table 1**. There was an equal number of males and females, but more males in group I (p=0.076). The mean age of the patients was significantly higher in group I (moderate to severe disease). In group I, fever was the most common symptom (80%), followed by cough (31.4%), shortness of breath (28.6%), and sore throat (8.5%). Chest pain and GI symptoms were present in 5.7% each. One patient (old treated case of pulmonary tuberculosis) had recurrent hemoptysis at presentation and another (known case of alcohol-related chronic liver disease) had altered sensorium due to hepatic encephalopathy. In group II, 67.3% were asymptomatic. Among those with symptoms, sore throat was the most common (52.9%) followed by cough (29.4%). Biochemical analysis revealed significantly higher leucocytosis with lymphopenia, hypokalemia, renal function parameters, and transaminases (**Table 2**). A significantly higher proportion of patients had hyperferritinemia, elevated D-dimer, and higher LDH, CRP, and procalcitonin in group I.

**Table 1:** Clinical parameters of disease severity in patients with COVID-19.

Parameter	Moderate to severe disease (n=35)	Mild disease (n=49)	p value
**Age (years)**	**53.5 ± 14**	**31.9 ± 13**	**0.000**
Gender (Males) (%)	62.9	40.8	0.076
**Fever (%)**	**75.9**	**31.4**	**0.000**
**Asymptomatic (%)**	**20**	**67.3**	**0.000**
**Duration of symptoms (days)**	**6 (3-9)**	**2 (1-7)**	**0.019**
**DM (%)**	**33.3**	**–**	**0.000**
**HT (%)**	**45.7**	**–**	**0.000**
PR (/min)	88.2 ± 11.8	87.5 ± 12.2	0.817
**SBP (mm Hg)**	**128.4 ± 19.0**	**114.7 ± 13.3**	**0.002**
**DBP (mm Hg)**	**80.3 ± 12.1**	**73.0 ± 9.0**	**0.008**
**PP (mm Hg)**	**48.1 ± 10.4**	**41.6 ± 9.3**	**0.013**
**MAP (mm Hg)**	**74.9 ± 12.6**	**66.0 ± 9.9**	**0.003**
**RR (/min)**	**22.2 ± 5.1**	**18.8 ± 2.3**	**0.001**
**FiO2**	**0.33 ± 0.17**	**0.20 ± 0.00**	**0.000**

Data are expressed as mean ± SD or median (q25-q75), as appropriate.

DBP, Diastolic blood pressure; DM, Diabetes mellitus; FiO2, Fraction of inspired oxygen; HT, Hypertension; MAP, Mean arterial pressure; PR, Pulse rate;, PP, Pulse pressure; RR, Respiratory rate; SBP, Systolic blood pressure. The bold values refer to the parameters that were significantly different between both groups.

**Table 2:** Biochemical parameters of disease severity in patients with COVID-19.

Parameter (Reference Range)	Moderate to severe disease (n=35)	Mild disease (n=49)	p value
Hemoglobin (g/dl)	11.6 ± 3.1	12.5 ± 1.9	0.171
(12-14g/dl)			
**TLC (x 10^9^/L)**	**9.5 ± 5.5**	**7.0 ± 2.9**	0.017
**(4.0-11.0 x 10^9^/L)**			
**Neutrophil (%)**	**72.0 ± 15.5**	**55.5 ± 11.8**	**0.000**
**(41-72%)**			
**ANC (x 10^9^/L)**	**7.3 ± 5.3**	**4.0 ± 2.3**	**0.005**
**(1.8-7.7 x 10^9^/L)**			
**Lymphocyte (%)**	**16.7 ± 11.0**	**32.9 ± 10.4**	**<0.001**
**(15-45%)**			
**ALC (x 10^9^/L)**	**1.2 ± 0.7**	**2.2 ± 0.9**	**<0.001**
**(1-4.8 x 10^9^/L)**			
Platelet count (x 10^9^/L)	182.2 ± 108.2	197.2 ± 83.1	0.563
(150-450 x 10^9^/L)			
**NLR**	**7.4 ± 5.9**	**2.0 ± 1.3**	**0.000**
**PLR**	**18.4 ± 12.4**	**9.9 ± 4.8**	**0.010**
Na (mmol/L)	141.6 ± 9.2	143.3 ± 3.6	0.484
(135-145mmol/L)			
K (mmol/L)	4.1 ± 0.6	4.3 ± 0.4	0.057
(3.5-5mmol/L)			
**Hypokalemia (%)**	**17.2%**	**–**	**0.011**
**(<3.5mmol/L)**			
**Urea (mg/dl)**	**40.2 (31.0-90.9)**	**22.6 (19.2-24.5)**	**0.004**
**(10-50mg/dl)**			
**Creatinine (mg/dl)**	**0.88 (0.78-1.15)**	**0.69 (0.55-0.84)**	**0.000**
**(0.5-1.2mg/dl)**			
BUN/Cr	22.7 (14.6-34.9)	18.9 (14.6-24.1)	0.347
Bilirubin (mg/dl)	0.51 (0.24-0.90)	0.39 (0.28-0.58)	0.522
(0.2-1.2mg/dl)			
**AST(U/L)**	**56.7 (45.7-85.7)**	**23.9 (19.3-44.4)**	**0.000**
**(2-40U/L)**			
**ALT(U/L)**	**61.6 (31.3-117)**	**29 (18.1-49.8)**	**0.002**
**(2-41U/L)**			
ALP (U/L)	92.5 (69.7-121.2)	96 (80-151.5)	0.512
(42-128U/L)			
**Albumin (g/dl)**	**3.26 (2.76-3.80)**	**4.42 (4.23-4.64)**	**0.000**
**(3.4-4.8g/dl)**			
**D-dimer (ng/ml)**	**794.5 (361.9-1954.2)**	**253.2 (148.4-470.7)**	**0.000**
**(0-240ng/ml)**			
**Elevated D-dimer (%)**	**91.7**	**55**	**0.002**
**(>240ng/ml)**			
**D-dimer ULN**	**3.31 (1.50-8.14)**	**1.06 (0.63-1.98)**	**0.000**
**Ferritin (ng/ml)**	**425.5 (171.0-813.9)**	**81.0 (33.7-155.5)**	**0.000**
**(30-400ng/ml)**			
**Hyperferritinemia (%)**	**66.7**	**33.3**	**0.000**
**(>400ng/ml)**			
**Ferritin ULN**	**2.91 (1.24-5.60)**	**0.54 (0.22-1.03)**	**0.000**
**LDH (U/L)**	**278 (186-398)**	**212 (172-271)**	**0.032**
**(135-225U/L)**			
**CRP (mg/L)**	**27.4 (6.08-77.09)**	**1.71 (0.47-5.79)**	**0.000**
**(0-5mg/L)**			
**CRP ULN**	**5.48 (1.21-15.42)**	**0.34 (0.09-1.14)**	**0.000**
**Procalcitonin (ng/ml)**	**0.12 (0.06-0.29)**	**0.03 (0.02-0.04)**	**0.000**
**(0.01-0.50ng/ml)**			
**HbA1c (%)**	**6.4 (5.5-7.1)**	**5.3 (5.0-6.0)**	**0.012**
**(<5.7% normal)**			
**CSS Category 2 or less**	**57.7**	**95**	**< 0.001**
**CSS category > 2**	**42.3**	**5**	

Data are expressed as mean ± SD or median (q25-q75), as appropriate.

- Nil (None of the patients with mild disease had hypokalemia).

The CSS (Scoring system of COVID-19) is a severity score developed and validated for patients with COVID-19. It includes variables like age, coronary heart disease, lymphocytes <8%, procalcitonin >0.15ng/ml, and D-Dimer >0.5µg/ml, with a cut-off exceeding 2 as associated with poor prognosis.

ALC, Absolute lymphocyte count; ALT, Alanine transaminase; ALP, Alkaline phosphatase; ANC, Absolute neutrophil count; AST, Aspartate transaminase; BUN, Blood urea nitrogen; CRP, C- reactive protein; CSS, Scoring system of COVID-19; K, Potassium; LDH, Lactate dehydrogenase; Na, Sodium; NLR, Neutrophil lymphocyte ratio; PLR, Platelet lymphocyte ratio; TLC, Total leucocyte count; ULN, Upper limit of normal. The bold values refer to the parameters that were significantly different between both groups.

**Figure 1:**
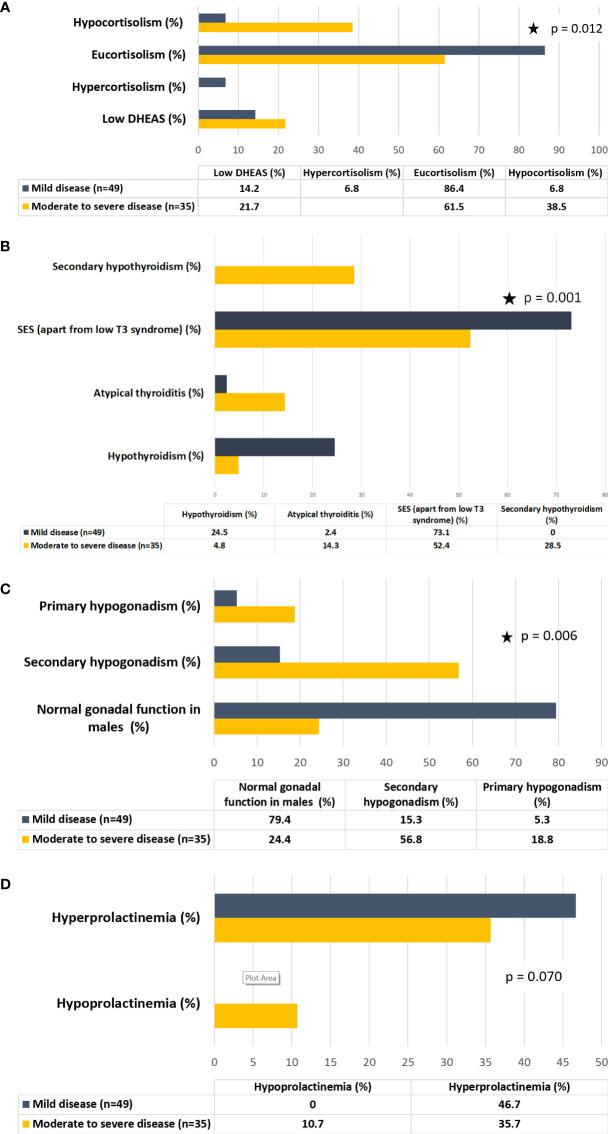
**(A)** Hypothalamo-pituitary-adrenal (HPA) axis dysfunction in patients with COVID-19 stratified on the basis of disease severity. **(B)** Hypothalamo-pituitary-thyroid (HPT) axis dysfunction in patients with COVID-19 stratified on the basis of disease severity. **(C)** Hypothalamo-pituitary-gonadal (HPG) axis dysfunction in male patients with COVID-19 stratified on the basis of disease severity. **(D)** Lactotroph dysfunction in patients with COVID-19 stratified on the basis of disease severity.

Parameters of endocrine dysfunction are summarized in **Table 3**. In group I, 38.5% of patients had hypocortisolism and the rest were eucortisolic among those who had paired hormone (cortisol and ACTH) values available (n=13), excluding patients like those mentioned above (**Figure 1A**). All hypocortisolic patients had low or inappropriately normal ACTH. Among those who were eucortisolic, 85.7% had normal or low ACTH, and 14.3% had high ACTH (>65pg/ml). In group II, 86.4% were eucortisolic, and 6.8% each had hypocortisolism and hypercortisolism. Among those who were eucortisolic in group II (n=40), 92.5% had normal ACTH, with only three having high ACTH. The lone patient with hypocortisolism in this subgroup had normal ACTH (38pg/ml). Among those with cortisol values exceeding 550nmol/l (n=3), only one had a high ACTH of 71 pg/ml. The majority of patients had normal DHEAS (78.3 *vs* 85.7%, p=0.49), whether eucortisolic or hypocortisolic.

**Table 3:** Comparative analysis of hormone levels between those with moderate to severe and mild COVID-19.

Parameter (Reference Range)	Moderate to severe disease (n=35)	Mild disease (n=49)	p value
Cortisol (nmol/l)	433 (353-571)	370 (279-454)	0.053
(170-550nmol/l)			
ACTH (pg/ml)	16.3 (11.3-53.2)	32.1 (21.7-44.8)	0.234
(5-65pg/ml)			
DHEAS (µg/dl)	86.2 ± 80.4	117.4 ± 62.0	0.086
(age and gender specific)			
Free T3 (pg/ml)	0.26 (0.21-0.38)	0.33 (0.30-0.35)	0.057
(2-4.4pg/ml)			
Total T3 (ng/ml)	0.63 (0.52-0.78)	1.11 (0.817-3.695)	0.119
(0.8-2ng/ml)			
Free T4 (ng/dl)	1.41 (1.14-1.56)	1.29 (1.17-1.45)	0.380
(0.9-1.7ng/dl)			
**Low free T4 (%)**	**9.5**	**–**	**0.020**
**(<0.9ng/dl)**			
**Normal free T4 (%)**	**76.2**	**97.6**	
**(0.9-1.7ng/dl)**			
**High free T4 (%)**	**14.3**	**2.4**	
**(>1.7ng/dl)**			
Total T4 (µg/dl)	5.14 (4.50-6.84)	4.98 (3.54-5.64)	0.913
(4-12 µg/dl)			
**TSH (µIU/ml)**	**1.48 (0.69-2.70)**	**2.64 (1.68-4.15)**	**0.003**
**(0.2-4.2 µIU/ml)**			
**Low TSH (%)**	**15.6**	**–**	**0.024**
**(<0.2 µIU/ml)**			
**Normal TSH (%)**	**75**	**77.8**	
**(0.2-4.2 µIU/ml)**			
LH (mIU/mL) (males)	8.7 (1.3-22.7)	6.3 (2.9-19.2)	0.136
(1.7-8.6mIU/mL in males 2.4-12.6mIU/mL in females)			
FSH (mIU/mL) (males)	2.65 (1.99-20.50)	3.8 (1.76-6.20)	0.474
(1.5-12.4mIU/mL in males 3.5-12.5mIU/mL in females)			
Testosterone (nmol/l)	2.93 (0.31-6.28)	7.50 (0.35-13.10)	0.079
(9-27nmol/l)			
LH (mIU/mL) (females)	18.7 (0.5-29.9)	7.4 (1.1-9.2)	0.124
FSH (mIU/mL) (females)	19.5 (1.8-26.6)	4.4 (1.5-6.2)	0.131
Estradiol (pg/ml)	36.8 (10.1-116)	68.6 (18.4-115)	0.455
Prolactin (ng/ml)	15.4 (8.7-26.6)	19.7 (14.6-36.6)	0.057
(5-20ng/ml in males 5-25ng/ml in females)			

Data are expressed as mean ± SD or median (q25-q75), as appropriate.

- Nil (None of the patients with mild disease had low free T4 or low TSH).

ACTH, Adrenocorticotrophic hormone; DHEAS, Dehydroepiandosterone sulphate; FSH, Follicle-stimulating hormone; LH, Luteinising hormone; T3, Tri-iodothyronine; T4, Tetra-iodothyonine; TSH, Thyroid-stimulating hormone. The bold values refer to the parameters that were significantly different between both groups.

With respect to the assessment of thyroid function, all patients (with entire thyroid function test values available, n=62) had low T3 syndrome in both groups. Low T4 syndrome was present in 9.5% of patients in group I, while none in group II had this syndrome. The absolute levels of free T3 were lower in group I (p=0.05) while there was no difference between free T4 levels in both groups (p=0.38). Overall, SES (apart from low T3 syndrome) was present in 80.9% of patients in group I and 73.1% in group II (**Figure 1B**). Typical thyroiditis was not observed in any patient in either of the groups. Atypical thyroiditis (defined as low free T3, high free T4, and normal/low TSH) was present more in group I than group II (14.3% *vs* 2.4%) (p= 0.046).

In group I, ACTH and T showed a moderate negative correlation with NLR (-0.447, p=0.042; -0.456, p=0.043). TSH showed a strong negative correlation with inflammatory parameters CRP (-0.547, p=0.004) and moderate correlation with hyperferritinemia (serum ferritin -0.460, p=0.014; ferritin ULN -0.465, p=0.015). In both groups, serum cortisol showed a moderate correlation with PLR (0.394, p=0.069 and 0.433, p=0.021). Both serum free T3 and free T4 showed moderate negative correlation with procalcitonin (-0.541, p=0.001; -0.435, p=0.009) in group II.

Patients in group I showed higher levels of LH and FSH as compared to group II (p>0.05). Testosterone levels were lower in group I, with moderate-to-severe disease (2.93 *vs* 7.50nmol/l, p=0.07). A significantly higher number of males in group II had normal gonadal function (78.9 *vs* 25%, p=0.006) (**Figure 1C**). Among those with gonadal dysfunction in group I, central hypogonadism was the more common pattern but primary hypogonadism was present in 18.8% of patients in group I and 5.3% of patients in group II. The prevalence of postmenopausal status was 15.4% in group I and 6.9% in group II. Serum prolactin was lower in group I, with 35.7% of patients having hyperprolactinemia and 10.3% of patients having hypoprolactinemia (**Figure 1D**).

## Discussion

The current study is the first, to the best of our knowledge, to comprehensively explore the prevalence, nature, and degree of endocrine dysfunction stratified based on disease severity at a dedicated COVID care center. We observed a higher prevalence of hypocortisolism, low ACTH, and DHEAS in patients with moderate-to-severe disease. Low T3 syndrome was a universal finding irrespective of disease severity. SES (apart from the low T3 syndrome) was more common in those with moderate-to-severe disease. A unique, less-frequently described pattern of atypical thyroiditis was present in 6.4% of the entire population, with greater prevalence in those with moderate-to-severe disease. Male hypogonadism was observed in a significant majority of those with moderate-to-severe disease, with secondary hypogonadism being more prevalent. Lactotroph function analysis showed a similar prevalence of hyperprolactinemia in both groups. These findings suggest that endocrine dysfunction is common and more severe in patients with severe COVID-19.

The dysregulated systemic inflammation characteristic of COVID-19 leads to severe multisystem pathology (26). We observed significantly greater renal and hepatic dysfunction, elevated ferritin, D-dimer, LDH, inflammatory markers, as well as a greater proportion of high CSS in those with moderate-to-severe disease. Patients with moderate-to-severe disease were significantly older and showed a male gender predilection.

Lessons from past experience with SARS, have taught us that multiple endocrine axes can be affected, manifesting as delayed central hypocortisolism and hypothyroidism and thyroid follicular cell injury (21–23). Due to the etiopathogenetic similarity between SARS-CoV and SARS-CoV-2, endocrine involvement in COVID-19 is likely to be present. But, it is imperative to acknowledge the multiplicity of involvement within a single endocrine axis. For example, thyroid function can be influenced directly by the virus, SES, HPT dysfunction, or any combination of these, resulting in a varying clinical picture. Similarly, the HPA axis may be involved at the central level (hypothalamus and pituitary) or at the level of adrenals due to hemorrhage/infarction and the HPG axis may be involved at the central level or direct viral-mediated cytotoxicity to the gonads owing to high expression of ACE2. Therefore, studies aimed to explore the integrity of the entire endocrine axis are essential, so as to improve understanding, with possible potential ramifications for therapy in the overall management of a COVID-19 patient.

### HPA Axis Function Assessment

In the current study, we observed a higher prevalence of hypocortisolism (38.5%) in those with moderate-to-severe disease, whereas nearly 86.4% of patients with mild disease were eucortisolic. Paired cortisol and ACTH, DHEAS analysis suggested that the predominant pattern of hypocortisolism was central hypoadrenalism. Further, in eucortisolic patients, the predominant pattern was a low or inappropriately normal ACTH, indicating insult to the corticotropes.

The basis of adrenal dysfunction stems from the observation of hypocortisolism in 39.3% of patients (n=61) in a cohort of SARS survivors (21). Concurrent thyroid dysfunction was present in 10% of patients, suggesting hypophysitis or hypothalamic dysfunction. Further, molecular mimicry between SARS-Co-V and peptide fragments of ACTH points towards the role of anti-ACTH antibodies (27). A similar strategy may be utilized by SARS-CoV-2, and exploring this possibility in the sera of affected patients can be useful. However, in an earlier study reporting cortisol assessment in COVID-19 patients, the median levels of cortisol were higher (619nmol/l) than in patients without COVID-19 (519nmol/l), but both groups were eucortisolic in terms of HPA axis evaluation during stress (8). Lack of reporting of concurrent ACTH and DHEAS levels and comparison with non-COVID patients was performed without information on disease severity, preventing analysis of the entire axis integrity. Our observation of secondary hypoadrenalism especially in those with moderate-to-severe disease suggests the possibility of hypophysitis as an underlying etiopathogenic mechanism in these patients. Alternatively, a relative increase in cortisol due to reduced clearance and acquired glucocorticoid resistance at GR-α during the initial stages of the neuroendocrine stress response will inhibit ACTH, which mimics a profile of functional central hypoadrenalism (28, 29).

The atypical finding in the current study was that DHEAS levels were normal, as opposed to expected lower levels in accordance with the progression of adrenal insufficiency (30). Usually, there is a dissociation between the rates of cortisol and DHEAS production, such that cortisol output is maintained till later during the course of illness by diverting the intermediates from adrenal androgen synthesis to the formation of glucocorticoids including cortisol. However, there is a small proportion of patients reported with normal DHEAS in presence of secondary adrenal insufficiency (21, 31). Additionally, DHEAS levels have been found to increase as an adaptive response in otherwise normal individuals exposed to acute stress (32, 33). This may be a plausible reason for our observation of normal DHEAS in the current study, as DHEAS estimation was performed within 24 to 48 hours of hospital admission.

### HPT Axis Function Assessment

We noted a range of patterns varying from new-onset hypo to hyperfunctioning of the HPT axis in COVID-19. Nearly three-fourths of patients with mild disease were euthyroid as compared to only half of patients with moderate-to-severe disease. Low T3 syndrome was not discriminatory as it was present in all patients. SES (apart from low T3 syndrome) was equally prevalent in both groups. However, the exceptional pattern of thyroid dysfunction that emerged in the current study included atypical thyroiditis in 6.4% of the entire cohort, with higher prevalence in those with moderate-to-severe disease.

The major pattern of HPT axis function in acute illness includes cytokine-induced SES (low T3 in mild illness, low T4 with increasing illness severity) with low or normal TSH (34). The other pattern of SES can be normal T4 with low TSH. Rarely, SES with high total T4 has been described, but mostly in patients with an underlying increased cause for thyroid-binding globulin (TBG) excess (35). Other factors that could influence the HPT axis in acute illness include alteration of TBG, use of drugs that can displace circulating T4 from TBG leading to high free T4 (e.g., heparin), and use of iodinated contrast for diagnostic imaging. In the current study, the issue of TBG alteration was circumvented by measuring free hormones and there was no/minimal exposure to contrast agents and heparin respectively. SARS-CoV-2 has been proposed to cause direct thyroid dysfunction owing to ACE2 mediated viral entry and destructive thyroiditis (15, 16). Though rarely, triggering of autoimmunity and Graves’ disease and myxoedema coma have also been reported in COVID-19 patients (18, 36).

In the current study, we observed a higher prevalence of thyroid dysfunction than reported in the THYRCOV study, possibly due to the inclusion of participants with moderate-to-severe disease (12). We noted a good negative correlation of TSH with CRP and ferritin. But, low free T3 was not observed in that study, while in another study assessing thyroid function in COVID-19 patients, lower total T3 and TSH were documented as compared to non-COVID-19 pneumonia and normal patients, without a significant difference in total T4 levels (13). We observed a similar state of low T3 syndrome in accordance with the fact that T3 is reduced even in mild disease severity, with increased conversion of T4 to reverse T3.

However, the most intriguing aspect of HPT axis assessment in our study was the observation of a subset of patients exhibiting a mixed pattern of thyroid dysfunction (atypical thyroiditis), possibly due to combined systemic inflammation at the level of the gland (destructive thyroiditis) as well as the pituitary (a component of SES). This is only the second report in the available literature to explore and validate this unique pattern of HPT involvement in COVID-19 (14).

### HPG Axis and Lactotroph Function Assessment

The gonadal axis in the current study, evaluated only in males, revealed a significantly higher prevalence of hypogonadism and lower levels of testosterone in those with moderate-to-severe disease. Further, testosterone levels were negatively correlated with NLR, suggesting an association between disease severity. More patients had secondary hypogonadism despite no greater prevalence of hyperprolactinemia than those with mild disease, suggesting a direct HPG axis suppression owing to the virus or cytokines. Interestingly, a three times higher prevalence of primary hypogonadism was also observed in those with moderate-to-severe disease with higher LH than FSH, suggesting a non-negligible proportion of patients with primary injury at the level of the gonad (especially Leydig cells) itself.

Gonadal function has been evaluated in patients with COVID-19 in a prior study which showed a primary testicular failure with significantly higher LH and a lower testosterone:LH ratio but no significant difference in testosterone levels (19). Our findings are similar to this study and suggest that primary testicular failure, especially Leydig cell insult, should be borne in mind in managing male patients with COVID-19.

Gonadal function in COVID-19 can be influenced by gonadal ACE2 expression leading to viral orchitis, hypogonadism due to cytokines inhibiting the HPG axis, use of glucocorticoids, or orchitis. Data on female gonadal function in COVID-19 are scarce. In the current study, we did not analyze detailed gonadal function since data on menstrual cycle details was not available in the majority of patients.

The strengths of the study include a comprehensive evaluation of all the components of various endocrine axes and the determination of the association of hormonal dysfunction with disease severity in COVID-19. Further, the exclusion of patients with variables that could affect HPA or HPT axes and estimation of free T3, T4 made the analysis more robust. The study has limitations, including a small patient number, cross-sectional nature, single time estimation, lack of a control group (including non-COVID pneumonia or patients undergoing major surgery with negative RT-PCR status), non-availability of adiposity measures or menopausal status, non-performance of parameters like cytokine profile and hormone-binding globulins, and non-evaluation of final clinical outcomes in enrolled patients. Though previous hormonal parameters were not available, patients with a known endocrine condition on treatment for the same were excluded from the analysis. Non-assessment of the short synacthen test (SST), anti-hypothalamic and anti-pituitary antibodies, MRI sella, nuclear scintigraphy of the thyroid, reverse T3 (which could have helped us to differentiate between sick euthyroid syndrome and secondary hypothyroidism) and thyroid autoantibodies, and evaluation of diabetes insipidus are other perceived limitations. The SST currently holds low accuracy in diagnosing hypocortisolism in acute stress, but we plan to follow-up patients for re-evaluation of hormonal axes and antibodies in sera in the future. This can aid in delineating whether it is the viral cytopathic effect or immunoinflammatory effect that is the predominant cause of endocrine involvement. Non-inclusion of a control group with non-COVID pneumonia or acute respiratory distress syndrome of similar severity to assess the COVID-specific involvement of the endocrine system is another limitation of the study. However, due to the limited sensitivity of PCR for diagnosing SARS-CoV-2 and the possibility that secondary bacterial infection in a given patient of COVID-19, rather than the viral disease per se, can cause endocrine dysfunction as well as COVID being the major focus of healthcare with resource constraints for other illness conditions, a control group was not included.

## Conclusion

Multiple endocrine organs and axes are potentially involved by the novel coronavirus SARS-CoV-2, with a greater prevalence of endocrine dysfunction in more severe disease. The involvement of multiple axes, particularly at the hypothalamo-pituitary level, suggests the possibility of hypophysitis as an underlying etiology. Follow-up surveillance of these patients at periodic intervals should be considered to unfold the etiology as well as the reversibility of dysfunction.

## Data Availability Statement

The original contributions presented in the study are included in the article/supplementary material. Further inquiries can be directed to the corresponding author.

## Ethics Statement

The studies involving human participants were reviewed and approved by Institute Ethics Committee, PGIMER. The patients/participants provided their written informed consent to participate in this study.

## Author Contributions

All authors contributed equally to the study and approved the final manuscript.

## Conflict of Interest

The authors declare that the research was conducted in the absence of any commercial or financial relationships that could be construed as a potential conflict of interest.
